# Supplementary material for: Prevalence of overweight and its associated factors among Malaysian adults: Findings from a nationally representative survey
Source: PLoS One. 2023 Aug 2;18(8):e0283270. doi: 10.1371/journal.pone.0283270 (PMC10395944; doi:10.1371/journal.pone.0283270)
Supplement: Attachment — Submitted filename: Response to Reviewers.docx [file pone.0283270.s001.docx]

Dear Editor & Reviewers,

We would like to express my sincere appreciation for taking the time to review and provide valuable feedback on our manuscript. Your thoughtful and constructive comments have been instrumental in improving the overall quality of the manuscript.

Your insights and expertise have helped us to identify the strengths and weaknesses of the manuscript, and we are grateful for your efforts in providing detailed feedback that has enabled us to address the concerns raised. Your comments have challenged us to think deeply about our research, and your suggestions have greatly enhanced the clarity and coherence of our arguments. Below are our responses for the comments:

1. Reviewer #1:

| No. | Reviewer comment | Response |
| --- | --- | --- |
| 1. | Please enrich your introduction with more references from literature, with reference to prevalence of obesity worldwide. | We have amended the text according to the constructive comment. |
| 2. | In the questionnaire section please include a reference for the questions used. Which instrument was used? | We have amended the questionnaire section to the data collection section, and this section will be explained in detail with respect to the independent and dependent variables. |

2. Reviewer #2:

| No. | Reviewer comment | Response |
| --- | --- | --- |
| 1. | better to mention the specific age range of the sample studied. | The study sample included individuals aged between 18 and 99 years old, and in accordance with Malaysia's guidelines, individuals who were 60 years old and above were considered as elderly. |
| 2. | the abstract should describe numerically the main results that are summarized while in the results section to add the specific p value next to the respective ranges of odd ratios discussed. | We have amended the text according to the constructive comment. |
| 3. | In the discussion, saying that the results are similar or on the contrary contrast with previous studies should be expanded to clarify more how. | We have amended the text according to the constructive comment. |

We are particularly grateful for the time and effort you have invested in reviewing our manuscript, and for the care with which you have considered the work. Your contributions have been invaluable, and we are grateful for your support throughout the review process.

Once again, thank you for your commitment in ensuring the highest standards of scholarly publishing. Your contributions are greatly appreciated, and we are honoured to have benefited from your expertise.
